# Spatiotemporal forecasting of opioid-related fatal overdoses: towards best practices for modeling and evaluation

**DOI:** 10.1093/aje/kwae343

**Published:** 2024-09-13

**Authors:** Kyle Heuton, Jyontika Kapoor, Shikhar Shrestha, Thomas J Stopka, Michael C Hughes

**Affiliations:** Department of Computer Science, Tufts University, Medford, Massachusetts, United States; Department of Data Science, Wellesley College, Wellesley, Massachusetts, United States; Department of Public Health and Community Medicine, Tufts University School of Medicine, Boston, Massachusetts, United States; Department of Public Health and Community Medicine, Tufts University School of Medicine, Boston, Massachusetts, United States; Department of Computer Science, Tufts University, Medford, Massachusetts, United States

**Keywords:** predictive modeling, spatiotemporal forecasting, machine learning, opioid, overdose, public health practice

## Abstract

To inform public health interventions, researchers have developed models to forecast opioid-related overdose mortality. These efforts often have limited overlap in the models and data sets employed, presenting challenges to assessing progress in this field. Furthermore, common error-based performance metrics, such as root mean squared error (RMSE), cannot directly assess a key modeling purpose: the identification of priority areas for interventions. We recommend a new intervention-aware performance metric, Percentage of Best Possible Reach (%BPR). We compare metrics for many published models across 2 distinct geographic settings: Cook County, Illinois, and the state of Massachusetts, assuming the budget to intervene in 100 census tracts out of 1000 in each setting. The top-performing models based on RMSE recommend areas that do not always reach the most possible overdose events. In Massachusetts, the top models preferred by %BPR could have reached 18 additional fatal overdoses per year in 2020-2021 compared to models favored by RMSE. In Cook County, the different metrics select similar top-performing models, yet other models with similar RMSE can have significant variation in %BPR. We further find that simple models often perform as well as recently published ones. We release open code and data for others to build upon.

## Introduction

The opioid overdose epidemic in the United States has resulted in over 450 000 deaths during the past 8 years, with more than 80 000 fatal opioid-related overdoses during 2022, the highest yet in a single year.^1^ Managing the opioid overdose epidemic requires a constellation of efforts ranging from substance use treatment programs offering medications for opioid use disorder,^2^^,^^3^ harm reduction programs, naloxone distribution, and comprehensive mental health and social support services.^4^^-^^6^ Beyond the provision of harm reduction and healthcare services, it is critical for policymaking to address the ever-evolving substance use environment and plan for targeted interventions.

There has been considerable variation in the availability of different types of opioids and the consequent increase in opioid use disorder and opioid-related fatal overdoses in the past 2 decades. The current fatal opioid overdose epidemic has been characterized by 4 waves.^7^^,^^8^ In the early 2000s, prescription opioids drove overdose deaths. Then, heroin-related deaths surged post-2010, followed by a fentanyl spike in 2013.^9^ This culminated with the fourth wave of combined stimulant and fentanyl-related overdose deaths.^8^ These shifts in supply accompanied changes in social and ecological conditions, impacting substance use behaviors differently across geographic regions.^10^^,^^11^ Hence, it is critical to examine local spatiotemporal variation in fatal opioid overdoses and predict future outcomes to inform preemptive public health responses.

A growing body of research^12^ has explored spatiotemporal variations in the opioid overdose landscape. Yet *forecasting* approaches are in a nascent stage, and there are few prediction studies at the population level.^13^ Other research focuses on patient-specific risk prediction,^14^^-^^16^ assuming access to detailed, person-level demographic and medical history data. Analyses focused on population-level predictions that solely depend on readily available aggregated data could be easily adopted by public health authorities with limited resources.

While several prior studies have identified historical overdose “hotspots,”^17^^-^^19^ fewer studies have forecasted future spatiotemporal overdose spikes. Research that focuses on hotspots often assumes that identified clusters are where the highest needs will exist in the future. In our analyses, we show that this assumption does not always hold. Existing research also spans a range of spatial and temporal resolutions. In geographic space, studies range from coarser county-level analysis^17^ to finer analyses based on ZIP Codes, census tracts, or census block groups.^20^ Temporally, studies range in focus across yearly aggregated,^17^ quarterly^21^, or weekly data.^22^

The overarching goal of our study is to help public health departments make short-term forecasts of future overdose events to enable planning of targeted interventions that are cognizant of limited resources. Our study focuses on forecasting for the identification of areas needing intervention, leaving evaluating the impact of intervention to future work. We forecast deaths, as these are an objective measure available across jurisdictions. Nonfatal overdose data are often difficult to obtain and may go unrecorded.

To understand the role of different forecasting models and evaluation metrics across communities and geographies, our evaluations cover 2 distinct catchment areas. First, we study Cook County, Illinois, covering over 5 million residents of Chicago and surroundings, where we forecast across 1328 populated census tracts from years 2015-2022. Second, we study the state of Massachusetts, with 1620 census tracts representing over 6 million residents from 2001-2021. These settings were selected based on data availability.

To establish best practices for modeling and evaluation, we carefully compare different modeling approaches and performance metrics in each area. We implement a comprehensive set of existing models—including heuristic baselines, statistical models,^12^^,^^20^^,^^23^ and neural networks.^22^ We assess the opioid-related fatal overdose forecasts they produce for both Cook County and Massachusetts at the census-tract-level at annual timeframes. We compute widely used error-based performance metrics and introduce a new intervention-aware performance metric. Our software is available for other researchers to reuse and build upon: https://github.com/tufts-ml/opioid-overdose-models.

## Methods

### Data sources and preparation

To assess models, we assembled 2 data sets suitable for forecasting opioid-related fatal overdoses annually at the census tract level. This study was reviewed by the Tufts University Health Sciences Institutional Review Board and deemed to be nonhuman subjects research.

Our relatively coarse annual temporal scale was chosen for 3 reasons: to match the frequency at which decision-makers might set new priorities; because final fatal overdose data has an approximately 6-month delay in Massachusetts; and because forecasting subannually is made much more difficult by increased sparsity. We chose the census tract spatial scale to reflect the urban nature of Massachusetts and Cook County, where intramunicipality variation is important for identifying intervention sites. In sparser jurisdictions, a coarser spatial scale may be appropriate. Each census tract contains a mean count of 4000 people (ranging from 1200-8000^24^). For many (not all) interventions, costs scale with population, and thus the cost of deploying an intervention in any tract is roughly uniform.

#### Data source 1: Cook County, Illinois

We obtained fully deidentified data from the Cook County Government Medical Examiner Case Archive^25^ for opioid-involved overdose deaths from August 2014 (the first date records are available) to May 2023. These data contained every fatal incident under the medical examiner’s jurisdiction that was determined to have any opioid as a primary cause. We used the provided incident latitude and longitude to map each fatality to one of 1328 census tracts. Because the data are in the public domain, we make our processed Cook County data available.

#### Data source 2: Massachusetts

We obtained death certificate data from the Massachusetts Registry of Vital Records and Statistics for opioid-involved overdose deaths between 2001 and 2019. These deaths were defined as unintentional, intentional, and undetermined drug poisonings containing an opioid code (ICD-10 codes T.40.0-T40.4, or T40.6) as a “multiple cause of death.” Each fatal overdose is linked to a calendar date and a residential street address. Decedent address is used, as the information for overdose location is incomplete in Massachusetts.^26^

### Data set preparation

For each data set, we computed the observed number of fatal overdose events *y_s,t_* at time *t* for individuals residing in spatial location *s*. We employed open tools to map locations (street address or latitude/longitude) to their corresponding census tract, using the tract boundaries for the states of Massachusetts and Illinois defined by the US Census Bureau in 2020.^27^

In each data set, a uniform set of covariates is available as input for prediction models. At each year *t* and census tract *s*, we provide the history of fatal overdose counts from previous times in that tract, as well as the geographic location (numerical latitude and longitude of the tract’s centroid) and timestamp (numerical time, measured in years since the first available year for that data set). Further, for each tract *s* at year *t*, an optional 5-dimensional covariate vector represents social vulnerability across 4 individual domains (socioeconomic status, age-related demographics, minority status, housing) plus a composite domain. Each value indicates that tract’s percentile ranking across all census tracts in that state for that domain, as published in the Social Vulnerability Index (SVI).^28^ Values are available for every census tract in the United States and updated every 5 years; we selected the closest value to each year. These SVI features were chosen for their simplicity, universal availability across US jurisdictions, and similar content as covariates in previous studies.^23^^,^^29^

### Metrics

To evaluate model forecasts against observed mortality, suitable performance metrics were essential. Our study considered both commonly used error-based metrics and a new intervention-aware metric.

#### Error-based metrics

Model performance is often assessed via summary statistics of the errors between predicted ($\overline{y_s}$) and observed mortality (${y}_s$) across all *S* spatial regions in the test period. Within this category, 2 common metrics are root mean squared error (RMSE) and mean absolute error (MAE), both defined in eqn 1. RMSE calculates the square root of the average squared errors, while MAE computes the average of absolute errors.


$$ RMSE=\sqrt{\frac{1}{S}{\sum}_{s=1}^S{\left({y}_s-\overline{y_s}\right)}^2}, MAE=\frac{1}{S}{\sum}_{s=1}^S\left|{y}_s-\overline{y_s}\right|\kern2.25em $$

Both RMSE and MAE have been used as the primary metrics to assess opioid overdose forecasting.^22^^,^^23^^,^^30^

#### Intervention-aware metric

In our intended use case, stakeholders at a public health agency could use a forecasting model to select a subset of census tracts in which to deploy an intervention to mitigate overdose deaths. We assume these actors have a limited budget, allowing intervention in a maximum of *K* of the *S* regions in their jurisdiction. For a given model, we can obtain its recommended set of *K* regions, which we refer to as the intervention set ***I***, in 2 steps. First, predict mortality counts for all *S* regions in the test period. Second, identify the *K* regions with the *K* highest predictions (breaking ties at random), and store these as the recommended set ***I**.*

To evaluate such recommendations, we need new metrics. The error-based metrics defined earlier (RMSE or MAE) aggregate error for all *S* regions equally. They do not directly measure if a forecast’s highest-risk areas specifically align with the actual areas of highest mortality. We thus wish to design a metric better aligned with how stakeholders determine intervention priorities. We suggest a model is favorable if the total count of fatal overdose events in its recommended set ***I*** of *K* tracts is as large as possible. This would indicate the model is good at identifying where adverse events will occur and thus increase the possibility that stakeholders could “reach” and hopefully mitigate these events via interventions targeted at the recommended tracts.

Our proposed metric, “best possible reach” (BPR), assesses a model’s recommendations via a ratio of 2 numbers, each one computed as a sum of a subset of the actual count vector $y=\left[{y}_1,\dots, {y}_s\right]$ of fatalities in the test period. First, the numerator counts how many actual fatalities occurred in the model’s recommended set ***I*** of size *K*. Second, the denominator counts the actual fatalities in the K regions that would be chosen with perfect hindsight of ***y***. Mathematically, we define BPR as,


\begin{align*} BPR&=\frac{\sum k\in{I}^{yk}}{\sum k\in TopKInds{(y)}^{yk}}\\& =\frac{actual\# events\ in\ K\ regions\ picked\ by\ model}{actual\# events\ in\ actual\ K\ highest\ count\ regions} \end{align*}

where *TopKInds(**y**)* denotes a function that returns the distinct indices of the *K* largest elements of vector ***y***. Appendix S1 illustrates computations of BPR on simple data sets, as well as a comparison to top-K adaptations of error-based metrics.

For public health applications, BPR is interpretable as the proportion of reachable fatal overdose events that interventions guided by the current model would reach. The BPR’s numerical value has a minimum of 0.0 and a maximum of 1.0. We typically convert the fractional BPR to a percentage ranging from 0%-100%, denoted as %BPR. A higher %BPR value signifies a more effective model at deciding where to intervene. A value of 100% indicates perfect decision-making given a limited budget: there is no other set of *K* regions any model could have recommended that would reach more events.

Although independently developed by our team (see our preliminary workshop paper^21^), our proposed BPR metric closely resembles the metric suggested by a recent pre-registered trial^31^ and a later feasibility study^20^ to evaluate opioid overdose forecasts in Rhode Island. The key distinction lies in the denominator: our BPR sums only the top *K* indices, while the alternative includes all *S* regions. We prefer our definition due to %BPR’s consistent range of 0%-100%. In contrast, the alternative’s maximum value fluctuates based on observed data in the test period, making it difficult to compare results across different time periods.

### Models

Below we define a range of possible forecasting methods that can be used for our where-to-intervene prediction task. All methods are trained and evaluated via a common protocol using the same provided splits (train/validation/test) of the 2 available data sets (Cook County and Massachusetts). Details about model fitting and hyperparameter tuning are provided in Appendix S2.

#### Simple baseline models

We study several easy-to-implement baseline models to highlight their comparative strengths. Public health practitioners seeking data-driven allocation of scarce intervention resources without sophisticated modeling could easily use these approaches.

Our first baseline, dubbed *all zeroes,* predicts uniformly across all *S* tracts that zero fatal overdoses will occur in the test period. This model, by definition, ranks all tracts as equally high risk, so for BPR which requires a set of *K* recommended regions we report an average over many samples of *K* distinct regions selected uniformly at random.

The other baseline we consider is a *historical average,* which predicts the next timestep’s mortality as an average of all mortality counts observed over the preceding *W* timesteps. Here, the appropriate “look back” period length *W* is the only hyperparameter.

#### Complex models

Next, we consider several more flexible models with parameters that can be fit to the data. The first is a generalized linear model (GLM) with a Poisson likelihood. This model assumes that fatal overdose count *y* for spatial tract *S* at time *t* is modeled by a Poisson distribution where the log of the mean parameter is a linear function of the covariate vector *x* for that tract and time:


$$ y \mid x,\theta \sim Poisson\left(\lambda ={e}^{\theta^Tx}\right) $$

We also include a Gradient Boosted Trees^32^ model, an ensemble of regression trees. Previous studies of opioid forecasting ^20^^,^^33^ have used similar tree ensembles.

We further include 3 spatially sophisticated statistical models used in recent opioid overdose forecasting applications. First, we include a Gaussian Process (GP) model^20^ for its ability to flexibly capture spatial and temporal correlations. We use similar covariance functions (“kernels”) to prior overdose forecasting work (details in Appendix S2). Next, Bayesian Spatio-Tempooral (BST) models^23^ use a Markov Random Field to model spatial and temporal trends. Thirdly, NBSpLag denotes a negative binomial regression model with spatially lagged features^29,^ where each tract is informed by its spatial neighbors. In a variable selection experiment,^29^ these spatially lagged covariates were found to be the most predictive features. Unlike previous evaluations of both BST^23^ and NBSpLag models, our study compares to the rich set of baselines described previously.

Finally, we include CASTNet,^22^ a neural network model designed for opioid-overdose forecasting. Unlike previous methods, CASTNet employs multihead attentional networks that allow predictions at a given location to be informed by other locations that are chosen in a data-driven fashion, not just selected by proximity.

### Experimental protocol

We applied each of the models described previously separately to the Cook County, IL, and the Massachusetts data sets. We used historical counts of opioid-related fatal overdoses (together with other covariates described previously) to predict future fatal overdose counts in each census tract and then assessed how these predictions can be used to recommend where to intervene.

For each data set, we assembled covariate vector, fatality count pairs $\left({x}_{s,t},{y}_{s,t}\right)$ for each year in the training set (*t* = 2010-2018 for Massachusetts, 2015-2019 for Cook County). The historical covariates inside each ${x}_{s,t}$ vector summarize the recent history of *W* previous years (*W* = 10 for Massachusetts, *W* = 5 for Cook County). Hyperparameters are chosen to maximize performance as assessed by BPR on a validation set of data from the year prior to evaluation (2019 for Massachusetts, 2020 for Cook County). Finally, models are evaluated on predictions for the final 2 years (2020-2021 in Massachusetts, 2021-2022 in Cook County).

From each model, we obtained predictions for each of the *S* tracts in each test year. We computed each evaluation metric in a way that quantifies our uncertainty to help us understand which differences are meaningful. Inspired by resampling methods for uncertainty quantification,^34^ for each test year we obtained 50 different subsets of 1370 of the 1620 census tracts in MA (1078 of the 1328 in Cook County, IL) by sampling without replacement. Sampling a distinct subset of tracts allows coherent assessment of how a method *ranks* distinct locations in need of overdose prevention interventions. We selected the number of tracts to preserve 85% of all fatal overdose events in an average sample (larger values led to samples that were too alike one another). For each sample, we compute RMSE and BPR and then report the mean as well as the min-max interval of the 50 samples.

In this set of experiments, we used a fine spatial scale (census tracts), a coarse temporal scale (yearly), and used a value of *K* = 100 census tracts. An intervention budget of *K* = 100 corresponds to the ability to intervene for approximately 400 000 people and is similar to *K* values used in other studies.^20^ We believe that this setting is well-suited to plan interventions such as naloxone distribution and opioid treatment programs,^35^ where planning with an annual timescale may be appropriate.^36^ The use of fine spatial resolution could be suitable for planning routes for mobile health services such as methadone or naltrexone mobile clinics and services for HIV and HCV counseling and testing.^37^ However, other choices of spatiotemporal resolution and intervention budget *K* may be better suited to other possible interventions.

## Results

Results from the experiments conducted on Massachusetts and Cook County data are summarized in Table 1 and Table 2, respectively. For further results including MAE scores, see Tables S1 and S2.

**Table 1:** Comparison of fatal opioid-related overdose prediction models trained on Massachusetts decedent data from 2010-2019, then evaluated on data from 2020 and 2021.

	**Metric**		
**Model**	**True total overdoses in top 100 tracts identified by model** ^**b**^	**%BPR (*K* = 100)** ^**c**^	**RMSE** ^**d**^
All zeros	124.1	25.1, (24.8-25.8)	1.92, (1.83-2.01)
Historical average (4 year)	295.4	59.8, (56.8-62.9)^a^	1.28, (1.24-1.33)
Poisson GLM + SVI	301.3	61.0, (57.4-65.1)^a^	1.38, (1.30-1.47)
Gradient boosted trees +SVI	293.6	59.5, (55.9-63.1)^a^	1.24, (1.18-1.30)^a^
GP	287.2	58.2, (55.2-61.2)	1.28, (1.23-1.34)^a^
CASTNet +SVI	268.0	54.4, (52.0-56.6)	1.47, (1.36-1.58)
BST + SVI	305.4	62.0, (60.0-63.7)^a^	1.23, (1.21-1.25)^a^
NBSpLag + SVI	305.4	62.0, (60.3-64.0)^a^	1.31, (1.27-1.35)

Abbreviations: BPR, Best Possible Reach; RMSE, Root Mean Squared Error; SVI, Social Vulnerability Index covariates; GLM, Generalized Linear Model; GP, Gaussian Process; NBSpLag, Negative binomial regression with spatially lagged features; BST, Bayesian spatiotemporal model.

^a^For each metric, the best model mean is indicated with an asterisk, along with any other model whose uncertainty interval overlaps this mean (intervals are computed via resampling methods).

^b^The column titled “True Total Overdoses in Top 100 Tracts Identified by Model” contains the *true* number of observed fatal overdoses in the top 100 tracts identified by the corresponding model.

^c^Here BPR (higher is better) is our new intervention-aware metric, computed assuming an intervention budget for *K* = 100 of 1620 possible tracts in Massachusetts.

^d^RMSE (lower is better) is a common error-based metrics.

**Table 2:** Comparison of fatal opioid-related overdose prediction models trained on Cook County, Illinois decedent data, from 2015 to 2020, then evaluated on data from 2021 and 2022.

	**Metric**		
**Model**	**True total overdoses in top 100 tracts identified by model** ^**b**^	**%BPR (*K* = 100)** ^**c**^	**RMSE** ^**d**^
All Zeros	137.0	21.7, (21.1- 22.4)	2.46, (2.34- 2.56)
Historical Average (4 year)	505.3	80.1, (76.2- 84.3)^a^	1.44, (1.38- 1.50)^a^
Poisson GLM + SVI	500.7	79.4, (76.0- 82.8)^a^	1.86, (1.77- 1.94)
Gradient Boosted Trees +SVI	355.7	77.1, (72.2- 81.9)^a^	1.55, (1.42- 1.68)
GP	477.2	75.7, (69.9- 80.5)^a^	1.63, (1.50- 1.74)
CASTNet +SVI	472.6	75.2, (73.2- 76.8)	1.53, (1.39-1.67)
BST + SVI	504.1	80.2, (78.7- 82.0)^a^	1.47, (1.43- 1.51)
NBSpLag + SVI	502.2	79.9, (78.1- 81.6)^a^	1.42, (1.37-1.48)^a^

Abbreviations: BPR, Best Possible Reach; RMSE, Root Mean Squared Error; SV, Social Vulnerability covariates; GLM, Generalized Linear Model; GP, Gaussian Process; NBSpLag, Negative binomial regression with spatially lagged features; BST, Bayesian spatiotemporal model.

^a^For each metric, the best model mean is indicated with an asterisk, along with any other model whose uncertainty interval overlaps this mean (intervals are computed via resampling methods).

^b^The column titled “True Total Overdoses in Top 100 Tracts Identified by Model” contains the *true* number of observed fatal overdoses in the top 100 tracts identified by the corresponding model.

^c^Here %BPR (higher is better) is our new intervention-aware metric, computed assuming an intervention budget for *K* = 100 of 1328 possible tracts in Cook County.

^d^RMSE (lower is better) is a common error-based metrics.

The best model(s) vary depending on the evaluation metric. We wish to assess how different metrics might lead to different priorities of where to intervene. To do this, we complete 2 steps in each catchment area. First, we identify the model(s) that would be preferred based on RMSE. Then, we report how many fatal overdoses were recorded in that model’s recommended set of *K* tracts and compare that to the corresponding number for a model chosen to optimize %BPR.

In Massachusetts, the Gaussian Process, Bayesian Spatiotemporal model with SVI (BST + SVI), and Gradient Boosted Trees + SVI all deliver top performance as assessed by RMSE. However, the BST has higher %BPR than the GP (62.0% compared to 58.2%). Interventions guided by the BST model preemptively identify 18 additional fatal overdoses per year compared to recommendations from the GP model.

In Cook County, there is no such gap in %BPR when comparing the *best* performing models by RMSE. However, we do observe that among some model pairs, the model with superior RMSE can have inferior %BPR. Gradient Boosted Trees with SVI covariates has superior RMSE to the GLM model yet worse %BPR (77.1% vs 79.4% for GLM). Interventions guided by the GLM model (preferred via the %BPR metric) could reach 15 more fatal overdose events annually than interventions guided by the Gradient Boosted Trees model with SVI covariates.

We emphasize that in several cases on both data sets, models selected using RMSE appear to perform as well as those selected by %BPR at the ultimate task of reaching the most fatal overdose events in the recommended set. While %BPR is not universally better at ranking models, we think this empirical evaluation demonstrates the value of reporting intervention-aware metrics such as %BPR alongside traditional metrics in order to select models that align best with downstream decision-making goals.

We also observe that while complex models like BST do well in both catchment areas, so does the simple *historical average* baseline. In Massachusetts, *historical average* delivers a BPR whose uncertainty interval overlaps the scores of best performing models. In Cook County, *historical average’*s uncertainty intervals overlap the best scores for both BPR and RMSE. The complex BST model here would reach less than 1 additional overdose annually than historical average.

## Discussion

Our study’s first contribution to the science of spatiotemporal forecasting of opioid-related overdose deaths highlights the need for extensive comparisons to a robust suite of simple baselines. This lesson matches reports^38^^,^^39^ from across the sciences, especially in health^40^^,^^41^ and the social sciences,^42^ that suggest advanced modeling techniques may not outperform simpler baselines on some prediction tasks. In each catchment area, across both intervention-aware and error-based metrics, we found that a historical average baseline performed competitively. If this simple model yields such high performance, it raises questions about adopting more complex counterparts that require specialized expertise. The simpler model should be considered unless there are compelling reasons to use complex models. Many prior overdose forecasting studies^20^^,^^23^ completely omit such baselines or include only the poor performing ones such as the last-year^29^ model or a too-long historical average.^22^ A key to success for this baseline is our data-driven selection of the number of years in the look-back period, following best practices for hyperparameter tuning.^43^^,^^44^ For all future studies of opioid overdose forecasting, we recommend including historical averages with tuned look-back periods.

Our second contribution, developed in parallel to contemporary work,^31^ is a new metric—percentage of best possible reach (%BPR)—which evaluates predictions based on their utility for informing decisions about where to intervene. We demonstrate that using BPR as an evaluation metric can lead to different model rankings and different recommendations of where to intervene than error-based metrics like RMSE. The net benefit of ranking models by BPR vs RMSE was neutral in Cook County. However, in Massachusetts, among the top-ranked methods by RMSE, using BPR improved by 18 the total annual fatal overdose events that could be preemptively identified. We believe that intervention-aware metrics like BPR more closely reflect how public health agencies wish to use forecasting models to inform their intervention strategies.^20^ We leave to future work the important question of predicting the impact of specific interventions.

While this study does not consider equity, BPR can be adapted to study equitable outcomes. As shown in other studies with similar metrics,^20^ BPR can reflect issues of health equity by requiring the inclusion of locations with desired geographic and demographic properties. The BPR also has applications in domains outside of public health such as disease confirmation or allocating sensors to observe wildlife.

Lastly, we emphasize that our study is designed to be reproducible and open to extensions by other researchers. We released the software for all models and metrics under a permissive open-source license. We also released our cleaned version of the public-domain Cook County data set alongside preprocessing code. Historically, overdose forecasting studies have not often shared code or data (reasonably due to privacy issues around decedent data). Enabling researchers to pursue a common prediction task via the availability of a public data set has been a key driver of progress in predictive modeling.^45^

### Limitations

This study has several limitations. First, our findings come from only 2 places (Massachusetts and Cook County) and may not be generalizable to other public health jurisdictions. Cook County is predominantly urban, while Massachusetts is a large state with substantial urban, rural, and suburban areas. The spatiotemporal trends in opioid-related mortality could thus be dramatically different in these 2 geographies, necessitating different model rankings and intervention strategies. This study is also impacted by COVID-19 pandemic. Training data from prior to 2020 may not accurately reflect the opioid epidemic in 2020 and beyond. Finally, we acknowledge that not all Cook County deaths are reported to the Medical Examiner. The Medical Examiner’s jurisdiction covers homicides, suicides, accidents, or sudden unexpected natural deaths for cause-of-death determination. We do not anticipate this impacting our results much.

Second, there are limitations to our analysis of the proposed BPR metric. For simplicity, all results here assumed an intervention budget of *K* = 100 census tracts. Different *K* values may lead to different method rankings. Our suggested BPR metric is intended for identifying where to intervene to relieve high overall burden. However, it does not directly prioritize the rate of change. Interventions aimed to reduce risk in communities that are at very high risk but do not already have a high burden may not be identified using BPR.

Finally, other choices of covariates are possible. Our focus on a limited set of covariates, derived from the SVI of the American Community Survey, was an intentional choice to ensure the nationwide availability of these covariates. Some jurisdictions may possess useful data sources, such as emergency medical service calls, insurance claims, and linked administrative data sets.^46^ Including such covariates could enhance model performance.

## Conclusion

We compared overdose forecasting options to better predict future fatal opioid-related overdose spikes and inform future harm-reducing interventions. Our study suggests the value of intervention-aware metrics like %BPR alongside traditional error-based metrics. Our study also suggests that simple baselines like historical averages should be included in future analyses, as more sophisticated and expensive-to-train models may not substantially outperform these baselines. As the opioid crisis continues to evolve, we hope our findings and our open-source resources enable improved model comparisons and data-informed interventions that ultimately reduce the harm caused by overdose events.

## Supplementary Material

Web_Material_kwae343

## Data Availability

We share our code for all models and metrics, as well as our cleaned data and preprocessing code for Cook County at the following repository: https://github.com/tufts-ml/opioid-overdose-models.
