# Supplementary material for: Spatiotemporal forecasting of opioid-related fatal overdoses: towards best practices for modeling and evaluation
Source: Am J Epidemiol. 2024 Sep 13;194(6):1776–82. doi: 10.1093/aje/kwae343 (PMC12133270; doi:10.1093/aje/kwae343)
Supplement: Web_Material_kwae343 [file web_material_kwae343.zip › HeutonBestPracticesSupplementProof.docx]

**Title**: Spatiotemporal Forecasting of Opioid-related Fatal Overdoses: Towards Best Practices for Modeling and Evaluation

**Authors**: Kyle Heuton, Jyontika Kapoor, Shikhar Shrestha, Thomas J. Stopka, and Michael C. Hughes

[Appendix S1: BPR Metric Details 1](#_Toc1994587207)

[Figure S1: Illustrated BPR calculation 2](#_Toc1746599864)

[Example S1: Calculating BPR 2](#_Toc815551061)

[Example S2: Model is bad at ranking 3](#_Toc1606870825)

[Example S3: Model good at ranking, but bad at accurate prediction of counts 3](#_Toc906099266)

[Example S4: Model perfectly forecasts true top-K tracts, but overestimates others enough that rankings are terrible 3](#_Toc1624624526)

[Appendix S2: Experimental Details 3](#_Toc1836042317)

[Additional Data details 4](#_Toc266040387)

[Additional Model details 4](#_Toc365363969)

[Table S1 Comparison of fatal opioid-related overdose prediction models trained on Massachusetts decedent data from 2010-2019, then evaluated on data from 2020 and 2021. 7](#_Toc136322365)

[Table S2. Comparison of fatal opioid-related overdose prediction models trained on Cook County, Illinois decedent data, from 2015 to 2020, then evaluated on data from 2021 and 2022. 9](#_Toc1818195182)

[References 10](#_Toc1685399310)

## Appendix S1: BPR Metric Details

### Figure S1: Illustrated BPR calculation


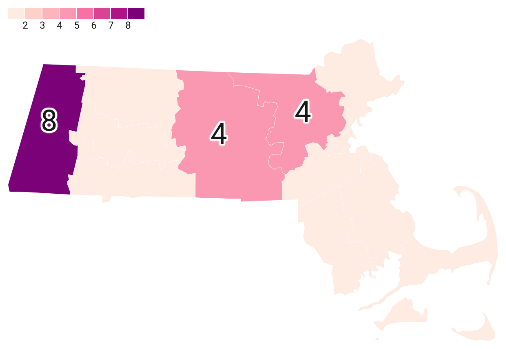

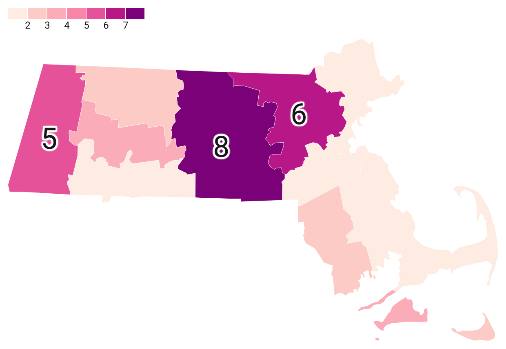

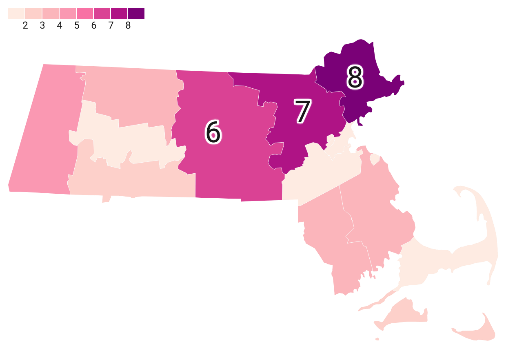
 A cartoon illustration of a BPR calculation for Massachusetts counties (simpler to visualize than using all 1620 census tracts). Here we demonstrate the BPR calculation for $K=3$. *Left*: ground-truth count values observed for the prediction period of interest. The top-3 true counties are labeled with their specific count values, and all other values are 1. The denominator of BPR will be 8+4+4=16. *Center*: A hypothetical forecast, attempting to predict the left map. Although the magnitude of every prediction is wrong, this forecast correctly identifies the top 3 counties and would achieve a Perfect BPR of 100%. *Right*: Another hypothetical forecast, but this time only two of the top-3 counties are correctly identified. To calculate the numerator of BPR, we sum the ground-truth counts from the predicted top-3 locations: 4+4+1=9, resulting in a BPR of 9/16=56.25%

### Example S1: Calculating BPR

Figure S1 shows a hypothetical scenario to demonstrate the calculation of BPR for the top-3 locations. The leftmost map depicts the synthetic "ground-truth." The top-3 counties are labeled. When calculating BPR, we always use the counts from the ground truth in both numerator and denominator. The model predictions alter which K locations we select for the sum in the numerator.

The denominator of BPR is the sum of the top-K locations in the ground truth data:

$$BPR = \frac{\sum_{k\in I} y_{k}}{\sum_{k\in TopKInds\left( y \right)} y_{k}}=\frac{\sum_{k\in I} y_{k}}{8+4+4}$$

Given the forecast in Figure S1 (center), the recommended top-3 locations ***I*** are exactly the same as the *TopKInds(y)* locations, so the BPR is perfect:

$$=\frac{8+4+4}{8+4+4}=100\%$$

Given the forecast in Figure S1 (right), the recommended top-3 locations ***I*** only contain two of the true *TopKInds(y)* locations. Again, to calculate the numerator, we take the ground-truth counts from the recommended locations, but this time they fall

short of a perfect BPR:

$$=\frac{4+4+1}{8+4+4}=\frac{9}{16}=56.25\%$$

**Why not evaluate MAE or MSE only on the top K predicted locations?**

It is natural to ask why not simply alter error-based metrics so they sum over the model’s recommended set of K locations, rather than all S locations. Below we consider several examples that highlight some of the tradeoffs involved with using BPR as a metric

### Example S2: Model is bad at ranking

| Location | 1 | 2 | 3 | 4 | 5 | 6 |
| --- | --- | --- | --- | --- | --- | --- |
| True Count | 2 | 2 | 2 | 12 | 12 | 12 |
| Prediction | **2** | **2** | **2** | 0 | 0 | 0 |

For this example, by design the model is poor at ranking tracts to identify those with the highest count. With *K*=3, the model would recommend tracts 1, 2, and 3, though ideally we would pick tracts 4, 5, and 6 as the true counts there are higher.

The top-K MAE metric cannot signal that this model is poor. The top-K MAE here is 0.0, essentially perfect for an error-based metric, with no further room for improvement. In contrast, our %BPR metric would score this model at 6/36= 16.7%, indicating plenty of room for improvement at ranking these tracts, since there always exists a tract ranking that would get 100%.

### Example S3: Model good at ranking, but bad at accurate prediction of counts

| Location | 1 | 2 | 3 | 4 | 5 | 6 |
| --- | --- | --- | --- | --- | --- | --- |
| True Count | 2 | 2 | 2 | 12 | 12 | 12 |
| Prediction | 12 | 12 | 12 | **22** | **22** | **22** |

For this example, by design the model is good at relative ranking, but poor at absolute magnitude estimation. Here, the top-K MAE is 10.0, indicating how even the top-K version of MAE is sensitive to magnitude issues, while our %BPR would be 100%, indicating how %BPR is insensitive to magnitude errors so long as the relative rankings are correct.

### Example S4: Model perfectly forecasts true top-K tracts, but overestimates others enough that rankings are terrible

| Location | 1 | 2 | 3 | 4 | 5 | 6 |
| --- | --- | --- | --- | --- | --- | --- |
| True Count | 2 | 2 | 2 | 12 | 12 | 12 |
| Prediction | **13** | **13** | **13** | 12 | 12 | 12 |

Here, the top-K MAE is quite poor, yielding a value of 11.0. The %BPR is also bad: 6/36 = 16.6%. Both metrics indicate subpar performance, as desired.

## Appendix S2: Experimental Details

### Additional Data details

For Cook County, we use the Medical Examiner’s provided defintion of “opioid related.” This consists of all deaths which have a primary or secondary cause containing one of the following terms: “ANPP, acetorphine, anaileridine, aplphaprodine, bromodol, buprenorphine, cinnamoyloxycodeinone, clonitazene, codeine, desomorphine, dezocine, dihydrocodeine, enadoline, etonitazene, etorphine, fentanil, fentanyl, heroin, hydrocodone, hydromorphone, kratom, meperidine, methadone, metopon, mitragynine, morphine, opiate, opioid, oxycodone, oxymorphone, pethidine, phenaridine, phenazocoine, phenomorphan, piperidine, spiradoline, tapentadol, tilidine, tramadol, and u47700.”^1^

To create the SVI covariates, we use a total of 5 variables. We use the 4 summary theme ranking variables: Socioeconomic Status, Household Characteristics, Racial & Ethnic Minority Status, and Housing Type & Transportation.^2^ We also use the overall vulnerability index, which is the ranked sum of the 4 summary themes. These are all percentile ranking values where a higher number indicates more vulnerability relative to other census tracts within the state.

### Additional Model details

Here we present additional details for all models in the main text, as well as several additional models excluded from the main text due to wordcount limitations. We present the full results for all models, along with RMSE calculated for each model.

Model hyperparameters are selected using the year prior to the test years as a validation year: 2019 for Massachusetts and 2020 for Cook County. When validating, models are trained through the year prior to the validation year. For evaluation on the test years, models are retrained through the validation year. Hyperparameters are selected by selecting the model with the highest BPR. In Massachusetts, we consider using up to 10 years of historical data when training (2010-2019). In Cook County there are fewer years of available historical data, and so training is limited to 2016-2020.

#### All-Zeroes

The All-Zeroes model is presented to highlight two things: the BPR of a naive policy, and the RMSE and MAE of a naive model. This model is very simple: every prediction is always 0 fatal overdoses. However, this presents a challenge for calculating BPR: what are the top K locations if every location is tied? In this case, we take 10,000 samples, and randomly pick the K locations to serve as the numerator for BPR. We then calculate the BPR for each of 10,000 samples, and report the average. By presenting a model with a prediction of 0 deaths, we also show the challenges with interpreting MAE and RMSE. All-zeroes is clearly a naive model, yet it is not immediately obvious what its MAE or RMSE should be.

#### Last Year

This model was omitted from the main text due to space constraints, but represents another simple baseline. Referred to as *last year,* this model predicts that the mortality at the next year in a specific location will mirror the mortality observed at the most recent recorded year for that location.

For this model, the prediction is simply the previous year’s fatal overdose count. When predicting for the second evaluation year (2021 in Massachusetts and 2022 in Cook County), the fatal overdose count from the first evaluation year is used. This is subtly different from the behavior of the regression models, where the models are trained using no data from the evaluation years.

#### Historical Average

In this model, the output is an un-weighted average of historical mortality. For both Massachusetts and Cook County 4 years are selected. These are both selected via the validation year. When predicting for the second evaluation year (2021 in Massachusetts and 2022 in Cook County), the fatal overdose count from the first evaluation year is used. This is subtly different from the behavior of the regression models, where the models are trained using no data from the evaluation years.

#### Weighted Historical Average

A description of the *weighted historical* average model was also omitted from the main text due to space constraints. This model is a weighted average of the previous W years of overdose events. This is more flexible than *historical average*, because each year’s count is multiplied by a customized weight coefficient.

This model is a linear regression on historical fatal overdose count using only past years mortality as a predictor. Scikit-learn’s^3^ ridge regression is used, which performs L2-regularized regression to minimize error in predicted fatal overdose count. The regularization strength α is selected via hyperparameter search by trying 29 evenly spaced values on a log scale between 10^-6^ and 10^8^. For Massachusetts, 10 years of historical data and an α of 10^4.5^ is used. For Cook County, 3 years of historical data are used and an α of 10^4.5^ is selected.

#### Linear Poisson GLM

This uses Scikit-learn’s^3^ Generalized Linear Model with a Poisson Likelihood and a log link. The hyperparameters explored are the number of prior years of mortality to include in the model and the L2 regularization strength α. Up to 10 years of previous mortality were considered for Massachusetts and 6 years for Cook County. The model is run with and without social vulnerability covariates. The model is trained to optimize the Likelihood of the observed fatal overdosessubject to the regularization penalty.

**Without social vulnerability covariates:** In Massachusetts 10 years of prior mortality are used in the model with an α of 1. In Cook County, 5 years of historical data are used with an α of 10^0.5^.

**With social vulnerability covariates:** In Massachusetts 6 years of prior mortality are used in the model with an α of 1. In Cook County, 5 years of historical data are used with an α of 10.

#### Gradient Boosted Trees

This uses Scikit-learn’s^3^ Histogram-based Gradient Boosted Trees model. We considered both squared-error and Poisson loss functions on the observed fatal overdoses. We tested using both 32 and 128 maximum iterations. For the minimum samples per leaf we used 9 equally spaced values between 2^0^ and 2^8^ on a log-2 scale. The maximum number of leaf nodes tested were 5 equally spaced values between 2^4^ and 2^8^ on a log-2 scale. Up to 10 years of previous mortality were considered for Massachusetts and 6 years for Cook County.

#### Gaussian Process Models

Here we use Scikit-learn’s^3^ Gaussian Process (GP) implementation. Due to the high computational cost of GP models, and following prior work[^4^](https://www.zotero.org/google-docs/?woSWdA), we only consider up to 5 years of historical data for both Massachusetts and Cook County, and we omit social vulnerability covariates. As in the prior work,we use a kernel that additively combines a Radial-Basis Function (RBF) kernel with a white noise kernel. The initial length scale of the RBF kernel is set to 0.5, and the noise level bounds on the white noise are set to (10^-5^, 10^1^). The outcomes are normalized to 0-mean and unit variance. The model is optimized to maximize the log-marginal likelihood of the given kernel function. Up to 9 restarts of the optimizer are used.

#### CASTNet

While we attempted to follow the original implementation of CASTNet[^5^](https://www.zotero.org/google-docs/?7Il8U1) as closely as possible, there are some significant modifications. The original CASTNet was concerned with predictions at a fine temporal-resolution, weekly. However, our initial experiments found little benefit at this scale, and we consider a much coarser scale: annual predictions. We lack the high-resolution crime data that the original CASTNet project uses as dynamic covariates. Furthermore, while we do have demographic and economic variables (the 5-dimensional Social Vulnerability index), these are not static at the annual scale but dynamic, accordingly these are used as the only dynamic covariates. For static covariates, only the latitude and longitude of the census tract are used. We use the hyperparameters selected by the original work: the LSTMs have a hidden unit size of 32 with a dropout value of 0.1, the group-level regularization coefficient is 0.0025, and the optimizer used was Adam with a learning rate of 0.5. The loss function consists of a component which penalizes errors in fatal overdose count, an orthogonality loss to ensure that the different “communities” identified by the model are distinct, and a Group Lasso regularization of the hyperparameters. Given that we have 2 evaluation years, we train the model twice, once with a lag time of 1-year and again with a lag time of 2-years. The 1-year lag model is used to predict for the first evaluation year (2020 in MA and 2021 in Cook County) and the 2-year lag model is used to predict for the second. This way no training data leaks into the model.

#### Bayesian Spatiotemporal Models

In the original work[^6^](https://www.zotero.org/google-docs/?DNzXXJ) on Bayesian Spatiotemporal models for opioid overdose forecasting, three separate models are proposed. All three models use an Autoregressive-1 term to model temporal dependence, and two of the models use spatial correlations. Because all three models are reported to behave similarly, we use what is called “Model 1”, lacking spatial correlations. Furthermore, the authors state that any number of temporal terms could be used, but do not specify which. Here, we choose a linear temporal term.This model is implemented using R-INLA[^7^](https://www.zotero.org/google-docs/?JOfUGM). Linear coefficients are used when adding the social vulnerability covariates.

#### Negative Binomial Regression with Spatially Lagged Covariates

The authors of this method[^8^](https://www.zotero.org/google-docs/?t26Vwt) helpfully provide code to run this model which we were able to use with little modification. Census tract level population estimates are taken from the same survey data as the social vulnerability covariates. The carrying capacity is initialized to 5% of the population in the first year of training data (2010 for MA, 2015 for Cook County).

## Table S1 Comparison of fatal opioid-related overdose prediction models trained on Massachusetts decedent data from 2010-2019, then evaluated on data from 2020 and 2021.

|  | Metric | | | |
| --- | --- | --- | --- | --- |
| Model | True Total Overdoses  in Top 100 Tracts  Identified by Model | %BPR (K=100) | MAE | RMSE |
| All Zeros | 124.1 | 25.1, (24.8-25.8) | 1.24, (1.18-1.30) | 1.92, (1.83-2.01) |
| Last year | 254.5 | 51.8, (49.5-54.4) | 1.09, (1.04-1.14) | 1.58, (1.50-1.65) |
| Historical Average  (4 year) | 295.4 | 59.8, (56.8-62.9)^*^ | 0.92, (0.90-0.94)^*^ | 1.28, (1.24-1.33) |
| Weighted  Historical Average | 303.5 | 61.4, (56.1-66.9)* | 0.94, (0.91-0.98)* | 1.32, (1.27-1.38) |
| Poisson GLM | 304.7 | 61.6, (56.4-66.8)* | 0.95, (0.91-0.98)* | 1.32, (1.25-1.39) |
| Poisson GLM +SVI | 301.3 | 61.0, (57.4-65.1)* | 1.10, (1.04-1.16) | 1.38, (1.30-1.47) |
| Gradient Boosted Trees +SVI | 293.6 | 59.5, (55.9-63.1)* | 0.92, (0.89-0.96)* | 1.24, (1.18-1.30)* |
| GP | 287.2 | 58.2, (55.2-61.2) | 0.93, (0.91-0.95)* | 1.28, (1.23-1.34)* |
| CASTNet +SVI | 268.0 | 54.4, (52.0-56.6) | 1.07, (0.99-1.16) | 1.47, (1.36-1.58) |
| BST | 301.0 | 61.1, (58.6-63.3)* | 0.95, (0.93-0.96) | 1.26, (1.24-1.28) |
| BST + SVI | 305.4 | 62.0, (60.0-63.7)* | 0.92, (0.91-0.94)* | 1.23, (1.21-1.25)* |
| NBSpLag + SVI | 305.4 | 62.0, (60.3-64.0)* | 0.92 (0.89-0.95)* | 1.31, (1.27-1.35) |

* For each metric, the best model mean is indicated with an asterisk, along with any other model whose uncertainty interval overlaps this mean (intervals are computed via resampling methods).

Here BPR (higher is better) is our new intervention-aware metric, computed assuming an intervention budget for K=100 of 1620 possible tracts in Massachusetts. MAE (lower is better) and RMSE (lower is better) are common error-based metrics. The column titled “True Total Overdoses in Top 100 Tracts Identified by Model” contains the *true* number of observed fatal overdoses in the top 100 tracts identified by the corresponding model.

Abbreviations: **BPR**: Best Possible Reach. **MAE**: Mean Absolute Error. **RMSE**: Root Mean Squared Error. **SVI**: Social Vulnerability Index covariates. **GLM**: Generalized Linear Model. **GP**: Gaussian Process. **NBSpLag**: Negative binomial regression with spatially-lagged features. **BST**: Bayesian spatiotemporal model

## Table S2. Comparison of fatal opioid-related overdose prediction models trained on Cook County, Illinois decedent data, from 2015 to 2020, then evaluated on data from 2021 and 2022.

|  | Metric | | | |
| --- | --- | --- | --- | --- |
| Model | True Total Overdoses  in Top 100 Tracts  Identified by Model | %BPR (K=100) | MAE | RMSE |
| All Zeros | 137.0 | 21.7, (21.1- 22.4) | 1.37, (1.30- 1.44) | 2.46, (2.34- 2.56) |
| Last year | 466.0 | 73.9, (70.7- 77.0) | 1.07, (1.03- 1.10) | 1.66, (1.59- 1.71) |
| Historical Average (4 year) | 505.3 | 80.1, (76.2- 84.3)* | **0.94, (0.90- 0.97)*** | 1.44, (1.38- 1.50)* |
| Weighted  Historical Average | 495.4 | 78.6, (75.7- 81.8)* | 1.15, (1.11- 1.19) | 1.87, (1.76- 1.96) |
| Poisson GLM | 496.8 | 78.8, (75.9- 82.3)* | 1.22, (1.08- 1.29) | 4.64, (1.65- 5.92) |
| Poisson GLM + SVI | 500.7 | 79.4, (76.0- 82.8)* | 1.16, (1.11- 1.20) | 1.86, (1.77- 1.94) |
| Gradient Boosted Trees +SVI | 485.7 | 77.1, (72.2- 81.9)* | 0.99, (0.95- 1.05) | 1.55, (1.42- 1.68) |
| GP | 477.2 | 75.7, (69.9- 80.5)* | 1.03, (0.97- 1.09) | 1.63, (1.50- 1.74) |
| CASTNet +SVI | 472.6 | 75.2, (73.2- 76.8) | 1.01, (0.98-1.04) | 1.53, (1.39-1.67) |
| BST | 505.9 | 80.5, (78.9- 82.1)* | 1.00, (0.98- 1.01) | 1.40, (1.36- 1.43)* |
| BST + SVI | 504.1 | 80.2, (78.7- 82.0)* | 0.97, (0.95- 0.98) | 1.47, (1.43- 1.51) |
| NBSpLag + SVI | 502.2 | 79.9, (78.1- 81.6)* | 0.96, (0.93-0.98)* | 1.42, (1.37-1.48)* |

* For each metric, the best model mean is indicated with an asterisk, along with any other model whose uncertainty interval overlaps this mean (intervals are computed via resampling methods).

Here %BPR (higher is better) is our new intervention-aware metric, computed assuming an intervention budget for K=100 of 1328 possible tracts in Cook County. MAE (lower is better) and RMSE (lower is better) are common error-based metrics. The column titled “True Total Overdoses in Top 100 Tracts Identified by Model” contains the *true* number of observed fatal overdoses in the top 100 tracts identified by the corresponding model.

Abbreviations: **BPR**: Best Possible Reach. **MAE**: Mean Absolute Error. **RMSE**: Root Mean Squared Error. **SV**: Social Vulnerability covariates. **GLM**: Generalized Linear Model. **GP**: Gaussian Process. **NBSpLag**: Negative binomial regression with spatially-lagged features. **BST**: Bayesian spatiotemporal model

## References

1. Medical Examiner Case Archive | Cook County Open Data. Accessed September 18, 2023. <https://datacatalog.cookcountyil.gov/Public-Safety/Medical-Examiner-Case-Archive/cjeq-bs86>

2. CDC ATSDR. Social Vulnerability Index 2018 Database for Massachusetts. Published online 2018.

3. Buitinck L, Louppe G, Blondel M, et al. API design for machine learning software: experiences from the scikit-learn project. Published online September 1, 2013. doi:10.48550/arXiv.1309.0238

4. Allen B, Neill DB, Schell RC, et al. Translating predictive analytics for public health practice: A case study of overdose prevention in Rhode Island. Am J Epidemiol. Published online May 17, 2023:kwad119. doi:10.1093/aje/kwad119

5. Ertugrul AM, Lin YR, Taskaya-Temizel T. CASTNet: Community-Attentive Spatio-Temporal Networks for Opioid Overdose Forecasting. In: Machine Learning and Knowledge Discovery in Databases: European Conference (ECML PKDD). ; 2019. Accessed September 23, 2022. <http://arxiv.org/abs/1905.04714>

6. Bauer C, Zhang K, Li W, et al. Small Area Forecasting of Opioid-Related Mortality: Bayesian Spatiotemporal Dynamic Modeling Approach. JMIR Public Health Surveill. 2023;9(1):e41450. doi:10.2196/41450

7. Rue H, Martino S, Chopin N. Approximate Bayesian inference for latent Gaussian models by using integrated nested Laplace approximations. J R Stat Soc Ser B Stat Methodol. 2009;71(2):319-392. doi:10.1111/j.1467-9868.2008.00700.x

8. Marks C, Abramovitz D, Donnelly CA, et al. Identifying counties at risk of high overdose mortality burden during the emerging fentanyl epidemic in the USA: a predictive statistical modelling study. Lancet Public Health. 2021;6(10):e720-e728. doi:10.1016/S2468-2667(21)00080-3
